# Developing Culturally Sensitive mHealth Apps for Caribbean Immigrant Women to Use During Pregnancy: Focus Group Study

**DOI:** 10.2196/humanfactors.9787

**Published:** 2018-10-10

**Authors:** Hana AlJaberi

**Affiliations:** 1 Department of Computer Graphics Technology Purdue Polytechnic Institute Purdue University West Lafayette, IN United States

**Keywords:** mHealth, human computer interaction, prenatal health, Caribbean, immigrant women, mobile phone

## Abstract

**Background:**

A valuable addition to the mobile health (mHealth) space is an exploration of the context of minorities in developed countries. The transition period postmigration, culture, and socioeconomic uniqueness of migratory groups can shed light on the problems with existing prenatal mHealth apps.

**Objective:**

The objectives of this study were to (1) use the theoretical concept of pregnancy ecology to understand the emotional, physical, information, and social challenges affecting low-income Caribbean immigrant women’s prenatal well-being practices and (2) develop a deep understanding of challenges worthy of consideration in mHealth design for these women.

**Methods:**

This qualitative interpretive approach using analytical induction presents the findings of 3 focus group sessions with 12 Caribbean immigrant women living in South Florida in the United States. The study took place from April to September 2015.

**Results:**

The participants revealed problematic tiers and support needs within the pregnancy ecology including emotional stressors caused by family separation, physical challenges, information gaps, and longing for social support.

**Conclusions:**

mHealth interventions for low-income Caribbean immigrant women must be designed beyond the conventional way of focusing on the events surrounding the unborn child. It can be tailored to the needs of the expecting mother. Pregnancy information should be customized on the basis of the variability of lifestyle, cultural practices, socioeconomic status, and social ties while still being able to deliver appropriate guidelines and clear cultural misconceptions.

## Introduction

### Background

Caribbean immigrants represent 9% of all immigrant populations in the United States [1]. More than half of the Caribbean immigrants are women. Pregnant women in the United States do not have their first medical visit until between 8-13 weeks into their pregnancy [2,3]. Immigrant women, in particular, do not initiate any type of medical visits because of additional access challenges, including the cost of health care, lack of health insurance, or its limitations [4,5]. Caribbean immigrant women face the risk of giving birth to very low birth weight children (<1500 g) at 2.4% compared with 0.7% for white American women [6]. In addition, they are susceptible to illnesses such as heart disease, asthma, poor breast health, and illnesses related to sexual intimacy [7]. Although their native tradition includes healthy foods, many are at risk of chronic type II diabetes for having to improvise their native diet in unhealthy ways when migrating to the United States [4]. The transition period postmigration can be especially challenging for the health of pregnant women. Added challenges for this minority group include limited knowledge of available medical resources in the new host country, lack of tailored care that considers culture and language barriers [5], and stigmas associated with seeking mental health [8]. Medical and information gaps in care lead immigrant women to tap into informal resources of information [4,9], which introduces incorrect, conflicting, and misguided information [3].

One way to address health disparities is through the use of mobile phones (mobile health, mHealth) as opposed to traditional means, such as pamphlets and brochures, which are impersonal and often easily lost and forgotten [2,3,10]. It can ensure the delivery of accurate and timely information along with support capabilities for better pregnancy management. Nonetheless, disparities in mHealth technology adoption are evident in lower-income minority populations in the United States because of the lack of tailored interventions [2,5]. Several models of mHealth, for example, use text messaging prompts [11], birthing chat rooms [3], and activity tracking with extensive input commands [12]. However, the impact of body changes and unpredictable energy levels during pregnancy might not be represented well through traditional designs of mHealth activity promoting tools. In addition, existing pregnancy mobile apps focus on topics surrounding the birthing event such as fetal development, countdowns to delivery, generic nutritional tips, and birthing complications [3]; it does not facilitate culturally and socioeconomically personalized information.

### Aims of the Study

During such vulnerable time, pregnancy can make women more receptive to interventions, which can arm them with health habits to extend beyond pregnancy [5,13-15]. The relationship between health outcomes for pregnant women and technology, in part through the creation of the term *pregnancy ecology* [3], seeks to create a nuanced understanding of the needs of pregnant women, who are often reduced to a series of data points and objectified as the adult carrying a baby to term. The term was specifically formed to emphasize the uniqueness of every pregnancy [3] and attempts to make clear the real value technologies can provide in aiding women with pregnancy health-related issues [2,3]. Therefore, the goal of this study is to develop a comprehensive understanding of the pregnancy ecology of low-income Caribbean immigrants that are deemed worthy of consideration in mHealth design.

## Methods

### Qualitative Study

This qualitative study adopted an interpretive paradigm that used an inductive analysis approach of its data collected from 3 semistructured focus group interviews. Focus groups were used to promote reflection among women for richer data [16] and for better time management with hard to recruit and busy participants. The focus group interview questions revolved around participants’ ideas of what it takes to have a healthy pregnancy, what they found challenging, and where they obtained their pregnancy information. The study was approved by the Human Research Protection Program’s Institutional Review Board at Purdue University.

### Recruitment

Recruitment occurred concurrently while conducting focus group sessions taking place in April to September 2015. Four women were enrolled per session, for a total of 12 participants distributed among 3 sessions. Recruitment was successful through the process of snowball sampling [17]. The study’s purpose and eligibility requirements were sent out via email to the contact list of Healthy Mothers Healthy Babies in West Palm Beach. In addition, they were posted on flyers along college and grocery store boards in South Florida and pitched in-person to potential participants through personal connections.

### Sampling

One way of strengthening rigor was recruiting a representative sample using a criterion-based sampling strategy where a predetermined set of criteria is used to identify appropriate participants for the goals of the study [17]. Eligibility criteria were (1) low-income Caribbean immigrant women living in South Florida, (2) able to communicate in English, (3) at least one full-term pregnancy between the ages of 18 and 30 and within the last 5 years in the United States, (4) familiarity with basic technologies such as the use of cell phones and the internet. No participant-specific demographic information was collected to make women feel comfortable and safe. The study involved a small sample size of 12 female participants partly because of the extensive effort and time it took to recruit and motivate participants to enroll and then schedule the sessions with them.

### Procedures

Each session took 1 hour to complete. The first 30 minutes were dedicated to reading the consent forms and familiarizing participants with the session rules. Each session was audiorecorded to help represent participants in this study accurately instead of relying on memory or the time-consuming process of note-taking. However, participants were advised that they could skip any question they do not want to answer or ask to stop recording at any time. A single copy of the audiorecordings was stored in a portable hard drive in an encrypted password-protected file that was destroyed within 2 months of transcribing the audiorecordings. No personally identifying information was audiorecorded. At the beginning of each focus group session, participants were asked to pick a fictitious name by which the moderator and other participants can use to communicate with them while the audiorecordings are in effect. After that, the focus group interviews took an average of 30 minutes to complete. Participants were asked to reflect on their pregnancy experiences as immigrants related to the following keywords: pregnancy, relationships, and technology (Multimedia Appendix 1). In addition, the questions provoked discussions of the important relationships influencing their pregnancy understandings, the technologies they used during pregnancy, and technologies used to interact with relationships affecting their pregnancy; furthermore, these questions probed participants to talk about their values indirectly. This was achieved by asking participants about common everyday events, obstacles, and behaviors in which they engage and which are important to them[18]. Even though this research focused on preventative mHealth care interventions, it did not lend itself to investigating a specific wellness initiative in prenatal health care for immigrant women. Per examination of previous research [3], women were more receptive to discussing their subjective perceptions of prenatal health versus discussing objective medical initiatives such as healthy weight gain during pregnancy.

### Data Analysis

The author transcribed audiorecorded focus groups immediately following each session, and an initial set of codes was developed. At that time, each transcription was supplemented with notes detailing the researcher’s initial reflections on possible themes. After that, transcripts were reviewed, compared, and coded using inductive constant comparative method [19-21], and then coded using an iterative coding process. Inductive coding was used to summarize the raw data into key themes that emerge from the data itself as opposed to being implied beforehand. Within each transcript session, the researcher used tentative short descriptive codes to describe excerpts of text. The process of rereading the transcripts and refining the descriptive codes was repeated several times. As subthemes emerged, the researcher consulted with past literature [3] to help make sense of them. Then, the researcher tried to establish connections between the subthemes. Later, these subthemes were combined into a broader theme. For example, subthemes such as family separation and abandonment were combined with the broader category of emotional stressors as the headline theme. All transcriptions and a rough draft of chosen quotes, paraphrased materials, and analysis were made available to the participants for review. Therefore, participants were given an opportunity to clarify any misrepresentation, and approve of the accuracy of the data as an approach to strengthen the rigor of this qualitative research through respondent validation. Of note, this work is also part of a dissertation paper that involved the cross-checking of analysis and interpretation strategies by committee members.

## Results

### Overview

**Figure 1:**
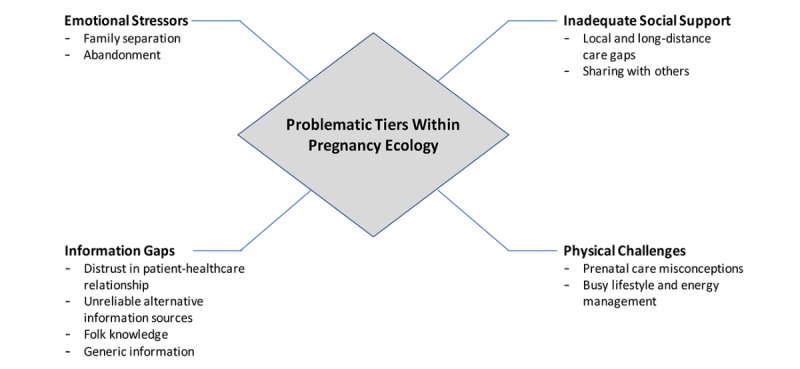
Problematic tiers within pregnancy ecology.

The following sections reveal themes and subthemes of problematic tiers and support needs within the women’s pregnancy ecology (Figure 1). Portions of the transcripts are quoted in numbers that reference either focus group 1, 2, or 3 and then either 1, 2, 3, or 4 for each of the participants within each focus group. For example, Participant 2.3 refers to participant number 3 within focus group 2.

### Emotional Stressors

This section aims to understand the emotional needs and stressors influencing the immigrant women’s ability to engage in health-promoting behaviors.

#### Family Separation

Acclimating to a new country during pregnancy is challenging because of the absence of family and social support system to lift some of the burdens during this time. Participants described pregnancy to be a family affair, as illustrated in the following quote:

The pregnant journey for me is about family, the family connection. With mother, sisters, cousin friends, and friends. We talk about it, we plan it together, we make decisions together, you need each other.

Participant 2.3

Consequently, women endured emotional stress during pregnancy triggered by homesickness.

#### Abandonment

Migration is a challenging time that is aggravated further during pregnancy, causing tensions between a woman and her partner. According to participants, pregnancy is celebrated among female members in Caribbean cultures. Despite such dynamic, separation from family support imposed lifestyle changes that require the expecting father to adapt and contribute.

When that does not happen, participants are left feeling abandoned and neglected, as illustrated in the following quote:

My husband like a ghost. He drink his beer and watch the tv without lifting na finger to help. Typical man…he can’t be bothered. I don’t need him anyway. What a man know? Na cook, na clean, na watch his own children to help me.

Participant 3.1

The resulting emotional stress and added burdens on their time leaves the pregnant women without the right frame of mind to pursue available prenatal resources and good dietary and fitness behaviors. However, participants with an understanding partner found pregnancy to be an easier process. A supportive partner predicts their mood and takes care of house chores so that they may rest or have time to pursue health activities, as illustrated in the following quote:

My husband know when I am pregnant is his turn to get the girls ready in the morning for school. Its more for him because if I get me time I am in better mood because when you pregnant you know you can loose it sometimes in the head.

Participant 2.2

### Physical Challenges

This section addresses the physical challenges influencing the immigrant woman’s prenatal health-seeking perceptions and behaviors.

#### Prenatal Care Misconceptions

During the focus group interviews, there were debates over several circulating prenatal guidelines. For example, there was a collective agreement among participants that deemed diet as one of the pillars to a successful pregnancy. However, there was confusion about appropriate diet and fitness guidelines. One major debate is the idea of eating for two without accountability, as illustrated in the following quote:

You eat for the baby and for you. Right now to be anorexic and worry too much about looking like supermodel better wait.

Participant 1.3

Another participant objected, as illustrated in the following quote:

You should eat your craving but in moderation. You should not want to be skinny of course, but you don’t just eat everything like you never going to have cake again. No, I am sorry, not good obviously.

Participant 1.2

Some women believed that such control could lead to a birthmark deformity and, therefore, the expecting mother must submit to the demands of her pregnant body, as illustrated in the following quote:

…if you don’t eat what you crave, your baby will have the blue with green marks somewhere in the body…I have cousins like that because my aunt man didn’t eat what she was craving. You don’t want your baby to live like that.

Participant 1.1

In addition, there was debate over appropriate and safe levels of exercise. Several participants were not into the idea of engaging in exercises once the physical appearances of pregnancy started to show, as illustrated in the following quote:

…walking is very good but not exercise especially when you start to show. You need enough rest and sleep.

Participant 2.1

However, when one participant expressed approval over the benefits of fitness during pregnancy, as illustrated in the following quote:

People say is not good to exercise when you start to show. Before you show is ok? Really? I use to think the same but walks ok only. But you have all celebrities exercise when pregnant, so I am curious now ok? I Googled, and find out it is good for you. It will make your mood better, and delivery of your baby so much easier.

Participant 1.4

Another participant interjected with sarcastic disapproval, as illustrated in the following quote:

…so you are one of the crazy Instagram pregnant woman with six abs.

Participant 1.3

#### Busy Lifestyle and Energy Management

The women’s migratory circumstances imposed lifestyle changes such as limited financial resources, changing roles in the household, and feelings of abandonment. Thus, some struggled with fatigue due to demanding responsibilities during pregnancy, as illustrated in the following quote:

I heard about exercising but my feet hurt too much after work and then I have to cook and clean. My sister helps but I’m just so tired!

Participant 3.3

Despite struggling with energy management during pregnancy, very few women recognized the benefits of exercising to improve energy levels, as illustrated in the following quote:

I also like walking and squatting every day when I am pregnant. Sometimes I do it first thing in the morning before I go to work, gives me good energy. Or before sun go down after work. Helps me with stress and give me some energy to cook and spend time with my family before bed.

Participant 2.2

However, some expressed that it was challenging to stay active because of their busy schedule, as illustrated in the following quote:

Think about it. Some of us might have two jobs. This city is not made for walking. Back home you walk a lot to get from a to b. But here if you take the bus, commute can be more than one hour, you sit on your ass.

Participant 1.1

### Information Gaps

This section discusses the participants’ perceived access challenges to prenatal information as a result of their migratory lifestyle.

#### Distrust in Patient-Health Care Relationship

Participants felt rushed during hospital visits and described doctors as impersonal and nurses as rude and impatient, as illustrated in the following quote:

I ask the nurse at the clinic and she turn her nose up at me. The doctor don speak in a language I understand then push me out.

Participant 3.1

Others accused the health care system of being a scam because of overused and unnecessary tests, as illustrated in the following quote:

They tell you all these things you need that you don’t need, or something wrong with you to charge you for tests you don’t need.

Participant 2.2

#### Unreliable Alternative Information Sources

As participants felt abandoned by medical professionals, they tapped into informal resources like Web-based search engines, family members, and mom friends. Search engines such as Google provided a platform for self-guided help. In addition, Google provided a discrete element for private use, as illustrated in the following quote:

Sometimes some of the questions you have is embarrassing to ask your mom or doctors. So I just go on Google.

Participant 1.2

Even though helpful, the women found such mediums yield at times conflicting and overwhelming information, as illustrated in the following quote:

But, many times I get very stressed because there is too many opinions to choose from. Or sometimes the language is very medical.

Participant 1.3

Because of these disqualifying characteristics, participants turned to family members and social circles for help, as illustrated in the following quote:

My mom and my sisters. We all have children so we talk about it all the time and we share advice when anyone is pregnant.

Participant 1.4

#### Folk Knowledge

There were many culturally and socially informed health tales during the focus group discussions. One participant recalled an encounter with her mother, as illustrated in the following quote:

When she came to see me first time I was pregnant, she never been to our apartment before, right? She freaking because the floors are tile, naaa you can’t walk inside your house without shoes because having bare foot on the tile hurt the baby. Actually, the bedrooms she thought were hardwood so can’t walk on either. But, ma these are, you know what you call them, you know, laminate, right? Yea yeah man laminate. I’m just dying laughing, she don’t know the difference.

Participant 2.1

Another participant recalled a story from her mother, as illustrated in the following quote:

My mom even tell me to cook the meat rare because the blood help the baby grow. What?! Eat anything red like red fruits because it is good blood for the baby.

Participant 2.4

Participant 2.1 expressed disapproval of such folk discourses as illustrated in the following quote:

I love my mom, I don’t know what I would do without her ever especially when I get pregnant. But, just there are some times she really get on my nerve and stress me out because she still old school, like the thinking.

Participant 2.1

…while Participant 2.3 expressed approval and belief in such discourses:

How about eating spicy food? Is that not good for the baby? I believe when people tell me things like that.

Participant 2.3

#### Generic Information

Participants disliked generic pregnancy print, Web, and mobile apps that focus mostly on the unborn child and the birthing event versus meeting the health and well-being needs of the expecting mother, as illustrated in the following quote:

Most apps about the baby. But, what about me? Even when is about the baby, is out of touch, you know. I am homesick when pregnant, I need a flavor of home there. Otherwise, I am just bored. It has stupid things like your baby now is this fruit size. I also want things for me, how I can manage emotion, exercise, eat good, dress comfortable, lotion, spanx, whatever to help me have healthy baby and also feel good.

Participant 2.2

For these immigrant women, other dislikes stemmed from information resources being insensitive to their socioeconomic status and cultural practices. Some women acknowledged wanting to take care of their diet. However, they were discouraged because existing prenatal resources do not factor their socioeconomic challenges into dietary and nutritional guidelines. Their socioeconomic status affects their perceptions of what is realistically attainable, which, in turn, discourages them from pursuing a healthier lifestyle, as illustrated in the following quote:

Okay think about it. You can want to be healthy all you want, is just wishes. The real life is a different story. Eating healthy food is very expensive.

Participant 2.4

In addition, existing resources lacked sensitivity over their cultural food preferences, as illustrated in the following quote:

I don’t trust what dem website say. People are different. I need answers from my own people, that why I ask family. All dem white lady doin the yoga, drinkin the Starbucks, and eatin like them bunny rabbit nothing but vegetables and blogs. These apps don’t tell me na ting new! I dun need pictures of how a white lady baby grow in her belly! Me want rice and beans, that brown stew, and leave me be.

Participant 3.1

### Social Support

In this section, the perceived social support capability gaps by immigrant women in existing systems are discussed.

#### Local and Long-Distance Care Gaps

Participants pointed out specific members of their social circle as sources of support for their emotional or informational needs when dealing with pregnancy stressors. For example, participants used social media, video, and group texting technologies to maintain ties in their home country during pregnancy, as illustrated in the following quote:

I always communicate with my mom when pregnant, more than the usual. She stays in my country. So, its hard to talk on phone whenever I like. But, she knows how to use Internet now, we use Whatsapp and Skype whenever we can.

Participant 1.3

In addition, participants wanted to see the role of the husband addressed, as illustrated in the following quote:

Also, this just funny, but help women know how to get their husband more involved since some have issues with that.

Participant 2.2

In expressing why they were not the intended match for existing apps, one participant argued that these were mostly designed for American women who might relate to pregnancy differently, as illustrated in the following quote:

It’s good for them maybe because you find some cute things like special dates in pregnancy, when your baby gets fingers and whatever, kicks or what that kick means. For some woman, maybe pregnant is hard, so maybe it can help you connect with emotion with your baby that you don’t know him or her. It can make it more fun when you are feeling not so good, your body hurts. I’m thinking, maybe…The pregnant journey for me is about family, the family connection. With mother, sisters, cousin friends, and friends. We talk about it, we plan it together, we make decisions together, you need each other. I’m too busy with that side of things, making memories.

Participant 2.3

#### Sharing With Others

Participants were comfortable sharing with a tight circle of parents and siblings, followed by few very close friends. The same courtesy was not extended to other family members and acquaintances, as illustrated in the following quote:

Ultrasound only for my mother and sisters and very close friends. Not even for the rest of the family…no ultrasound for everyone to see. If you have haters, you need to be careful.

Participant 1.3

The findings revealed that the women’s sharing habits in their personal social media profiles proceeded with caution during pregnancy. One reason for such cautious, limited practices is cultural beliefs. Some believed that people’s jealousy or envy might cast a curse, leading to misfortune, and that some might inflict harm on you through acts of witchcraft and voodoo practices, as illustrated in the following quote:

I myself scared to share too much happy pictures because there are haters, people you know, and I don’t want something bad to happen to my baby.

Participant 1.1

When participants did encounter pregnancy Web or mobile tools, they recalled social capability features such as chat rooms that grouped women together with the same birth month. Participants disliked such tools and cited reasons of observing bullying incidents, receiving conflicting information, and responses that go out on irrelevant tangents, as illustrated in the following quote:

If you have a good question, no one answer, no one care. Only if you a drama queen question, like my baby daddy drama, I don’t know what.

Participant 2.1

A participant went so far as to describe such mediums as an episode of “bad girls club” [Participant 2.1], a reality television series of clashing personalities living under one roof.

## Discussion

### Design for the Expecting Mother

Participants did not find something specifically directed at their needs as expecting mothers other than generic pregnancy mobile and Web apps focusing on the relationship to the unborn child, birthing, and postbirthing events. These solutions fail to be relevant in addressing prenatal health care challenges faced by recent immigrant women. Several women acknowledged wanting to manage pregnancy and adopt a healthier lifestyle but admitted to not having the proper information and circumstances to do so. Therefore, the study advocates for interventions that focus on the expecting mother by providing support with immigrant women’s physical, information, social, and emotional stressors.

### Tailored Interventions

One crippling access challenge to prenatal medical information for the interviewed immigrant women is not mapping information to align with their demanding day-to-day lifestyle, and lacking sensitivity to their cultural practices. For example, dietary guidelines and nutritional suggestions do not consider minorities’ cultural connections [4,22-24] that affect their food preferences and choices. As the results revealed, there is a disconnection between medical information and the realities these women live. For immigrants, there is an emotional connection with familiar food, especially when acclimating to a new unfamiliar environment [4]. In addition, the study calls for sensitivity to the women’s low-income socioeconomic status when recommending nutritional guidelines. The case in point here is not solely over what the recommended cuisine should be. From a design perspective, the bigger picture is that participants would adopt technologies that support their lifestyle. Participants felt excluded from prenatal health tech solutions, perceiving them as designed exclusively for “white rich ladies [Participant 2.3].” Thus, interventions must deliver information in a way that supports their busy day-to-day lifestyle, cultural practices, and socioeconomic status to achieve successful practice and adoption.

### Clear Cultural Misconceptions

Because of medical access challenges, the immigrant women seek guidance from their social circles and the internet instead. Women are then exposed to conflicting, misguided, and false information. The debates reported earlier surrounded topics such as safe fitness levels and practices, ideal food consumption habits, and healthy weight management; these are similar misguided topics reported for low-income pregnant American women [3]. However, the study’s findings added cultural discourses unique to this demographic. In this case, examples included narratives linking birthmarks to unfulfilled cravings, red foods and baby animals for fetal development, wood floors to miscarriages, and so on. The design of mHealth interventions should address not only common misconceptions but also culturally and socially specific folk wisdom discourses specific to a targeted demographic. This is an area in which mHealth design can make a significant contribution to pregnant immigrant women’s health.

### Include Social Circles

The findings revealed the significant other, whether compassionate or indifferent, plays a major role in a woman’s pregnancy. Another example was revealed in the role of family and close friends play in an immigrant pregnant woman’s life. Because of the long-distance separating families and the idea that pregnancy is a family affair, facilitating a platform for the family to participate in an immigrant’s pregnancy practices presents an opportunity for mHealth design. While previous studies [3] emphasized the role of the spouse alone in a woman’s pregnancy, this study introduces the mother and siblings who are just as important. Mothers especially seem to play a dual role, in which they are a reliable support system, while at the same time a source for folk misconceptions. The mother’s role is something that has been ignored in past mHealth literature. Such relationships represent indirect stakeholders that might affect whether the woman decides to adopt a technology or not. Thus, designs should prompt others to participate in the intervention to aid in supporting the user. This type of interaction engages the intimate relationships in a pregnant immigrant’s life and provides a platform for rebuilding social support with relationships that affect their health behaviors. In previous health communication human-computer interaction and social networking research [25-27], pregnant women have been described as being comfortable sharing pregnancy and motherhood information on Web-based social settings, even with strangers; this certainly contradicts with the findings in this study and a previous study on low-income pregnant American women by Peyton et al [3].

The author takes a more in-depth view of this aspect in a recently published work [28]. The study expands the discussion on the social theme that emerged from the focus groups here and uses codesign workshops under a participatory action framework to propose social and organizational design needs and recommendations for effective mobile tools for the women. In addition, it explores the immigrant women preferred interaction scenarios in mHealth design.

**Figure 2:**
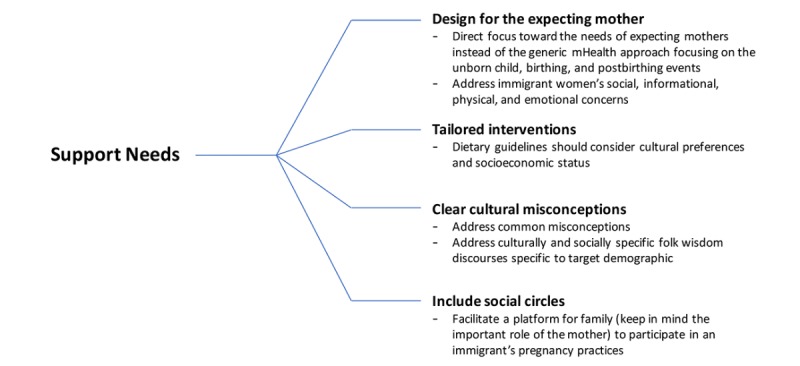
Design recommendations.

Stemming from the study’s findings, an alternative approach to designing prenatal mHealth technologies that can be explored and expanded further in future studies is set forth with the following recommendations: design for the expecting mother’s needs, design tailored interventions, clear misconceptions, and consider the role of social circles (Figure 2).

### Limitations

Those who could not communicate in English were excluded from the study. In that sense, the study did not take into account language barriers as part of a comprehensive picture of understanding the barriers to accessing available technologies. In addition, the small sample size in this study presents a challenge to the generalizability of the study findings. However, the inductive exploratory nature of this research warrants and benefits from the use of small sample sizes. A small sample size allows the researcher to assume an active role in recruitment and engagements with participants, which can help generate richer multidimensional data. It is also convenient to attain continual access with participants to validate the data and strengthen its reliability [29,30].

### Conclusions

Migration stressors can impose health challenges on a pregnant woman. As this study joins others in addressing the health needs of minority groups, it advocates designing of appropriate prenatal mHealth interventions that explore the multidimensional ecology of pregnant low-income immigrants. Thus, the study’s methods aimed to understand how immigrants view their ecological gaps that challenge and influence technology design and adoption. Prenatal mHealth interventions must be explored beyond the traditional way of focusing on the events surrounding the unborn child. They must tap into the needs of the expecting mother and beyond by, for example, considering the role social ties play as motivators or challengers to her pregnancy well-being. Furthermore, it must explore ways that customize pregnancy information based on the variability of lifestyle, cultural practices, and socioeconomic status, and yet be able to deliver appropriate guidelines.

## Appendix

Multimedia Appendix 1 A script of focus group questions.

## Abbreviations

mHealth: mobile health
