# Supplementary material for: Developing Culturally Sensitive mHealth Apps for Caribbean Immigrant Women to Use During Pregnancy: Focus Group Study
Source: JMIR Hum Factors. 2018 Oct 10;5(4):e29. doi: 10.2196/humanfactors.9787 (PMC6231776; doi:10.2196/humanfactors.9787)
Supplement: Multimedia Appendix 1 [file humanfactors_v5i4e29_app1.pdf]

ppendix I Procedures Script

Focus Groups:
Reflect on your pregnancy experiences as an immigrant related to the following

keywords (topics)?

**Keyword**	**Moderator add-on probes**
Pregnancy	• In your opinion, what does it take to have a healthy pregnancy?<br>• What was challenging during your pregnancy?<br>• How did you obtain your pregnancy information?
Relationships	• Whom do you most often communicate with in the US or your home country during your pregnancy?<br>• Can you describe the roles relationships with domestic partner, friends, and family play in your understandings of pregnancy?<br>• What are the strengths and weaknesses of each that might at times make one take precedence over the other?
Organizations	• Discuss the role of care providers and social or religious organizations whether in the US or your home country during pregnancy in the states?
